# Pretreatment Donors after Circulatory Death with Simvastatin Alleviates Liver Ischemia Reperfusion Injury through a KLF2-Dependent Mechanism in Rat

**DOI:** 10.1155/2017/3861914

**Published:** 2017-11-19

**Authors:** Zhongzhong Liu, Xingjian Zhang, Qi Xiao, Shaojun Ye, Chin-Hui Lai, Jun Luo, Xiaoying Huang, Wei Wang, Cheng Zeng, Zibiao Zhong, Xiaoli Fan, Zhiping Xia, Yan Xiong, Xinfang Mao, Qifa Ye, Yanfeng Wang

**Affiliations:** ^1^Zhongnan Hospital of Wuhan University, Institute of Hepatobiliary Diseases of Wuhan University, Transplant Center of Wuhan University, Hubei Key Laboratory of Medical Technology on Transplantation, Wuhan, Hubei 430071, China; ^2^Research Center of National Health Ministry on Transplantation Medicine Engineering and Technology, The 3rd Xiangya Hospital of Central South University, Changsha, Hunan 410013, China; ^3^Xinjiang Key Laboratory of Biological Resources and Genetic Engineering, College of Life Science and Technology, Xinjiang University, Urumqi, Xinjiang 830046, China

## Abstract

**Objective:**

Severe hepatic ischemia reperfusion injury (IRI) can result in poor short- and long-term graft outcome after transplantation. The way to improve the viability of livers from donors after circulatory death (DCD) is currently limited. The aim of the present study was to explore the protective effect of simvastatin on DCD livers and investigate the underlying mechanism.

**Methods:**

24 male rats randomly received simvastatin or its vehicle. 30 min later, rat livers were exposed to warm ischemia in situ for 30 min. Livers were removed and cold-stored in UW solution for 24 h, subsequently reperfused for 60 min with an isolated perfused rat liver system. Liver injury was evaluated during and after warm reperfusion.

**Results:**

Pretreatment of DCD donors with simvastatin significantly decreased IRI liver enzyme release, increased bile output and ATP, and ameliorated hepatic pathological changes. Simvastatin maintained the expression of KLF2 and its protective target genes (eNOS, TM, and HO-1), reduced oxidative stress, inhibited innate immune responses and inflammation, and increased the expression of Bcl-2/Bax to suppress hepatocyte apoptosis compared to DCD control group.

**Conclusion:**

Pretreatment of DCD donors with simvastatin improves DCD livers' functional recovery probably through a KLF2-dependent mechanism. These data suggest that simvastatin may provide a potential benefit for clinical DCD liver transplantation.

## 1. Introduction

Liver transplantation is the only successful life-saving treatment for patients with most types of end-stage liver failure [1]. However, the shortage of adequate organs all over the world has led to the use of extended-criteria donor (ECD) organs from steatotic donors or donors after circulatory death (DCD). Moreover, these DCD organs often suffer from an unpredictable longer warm ischemia time, which are much more prone to a higher risk of early allograft dysfunction (EAD) or primary graft failure (PNF) after transplantation [2, 3]. The unavoidable hepatic ischemia reperfusion injury (IRI) induced by warm and cold ischemia is one of the main reasons of the poor outcome after liver transplantation [4, 5]. Therefore, new concepts of drug and preservation have been suggested to improve graft viability and reduce hepatic IRI.

Kruppel-like factor 2 (KLF2) is a laminar flow inducible transcription factor primarily expressed by the endothelial cell and plays an important role in the regulation of endothelial function [6–8]. It induces factor expression of vasodilators and antithrombotic, antioxidant, and anti-inflammatory genes (e.g., endothelial nitric oxide synthase (eNOS), thrombomodulin (TM), and heme oxygenase-1 (HO-1)) and reduces the expression of adhesion molecules (vascular cell adhesion molecule 1 (VCAM-1) and E-selectin), conferring a vasoprotective endothelial phenotype [9–11].

Clinically, statins are antihyperlipidemic agents designed to reduce morbidity and mortality caused by cardiovascular diseases through lower cholesterol levels. In addition, statins have shown other vasculoprotective properties [12–14]. Recently, Barcelona team has demonstrated administration of simvastatin protecting the liver sinusoidal endothelium through upregulating KLF2 expression, both in experimental models of partial (70%) hepatic warm ischemia and cold ischemia-induced acute liver injury [15–18]. Nevertheless, the potential mechanisms of DCD liver pretreatment with simvastatin remain unknown.

Therefore, in the present study, the aim was to evaluate the effects of simvastatin pretreatment on DCD livers and explore the underlying mechanisms. We hypothesized that simvastatin could improve graft viability and attenuate liver IRI via decreasing the levels of proinflammatory cytokines and reducing hepatic oxidative stress and apoptosis, which might be related to the activation of KLF2.

## 2. Materials and Methods

### 2.1. Experimental Design

Male Wistar rats (250–300 g) were purchased from the animal experiment center of Wuhan University. All rats were maintained on standard laboratory water and chow according to the Experimental Animal Regulations of the People's Republic of China and the Guide for the Care and Use of Laboratory Animals. All rats were fasted for 12 h before the experiment. The 24 rats were randomly divided into 4 groups (*n* = 6 for each group). Rats were treated with simvastatin (1 mg/kg i.p., MedChem Express, NJ 08852, USA) or its vehicle (DMSO 0.1%) 30 min [18] before in situ warm ischemia. We established a rat DCD model [19], in which through incision of the diaphragm results in cardiac arrest without prior heparinization and portal clamping. The period of in situ liver warm ischemia started from the point of cardiac arrest. In the second step, the livers procured were stored for 24 h at 4°C in University of Wisconsin (UW) solution. In the third step, we evaluated reperfusion injury using the isolated liver perfusion model.

We chose the following experimental groups:

1. DCD simvastatin group (DCD-Sim group): simvastatin pretreatment, DCD livers exposed to 30 min of in situ warm ischemia followed by 24 h cold storage (UW solution) and subsequent 1 h ex vivo warm reperfusion.
2. Cold storage reperfusion group (CSP group): normal livers without warm ischemia, subjected to 24 h cold storage (UW solution) and subsequent 1 h ex vivo warm reperfusion.
3. DCD control group (DCD-Con group): vehicle (DMSO 0.1%) pretreatment (no simvastatin pretreatment), DCD livers exposed to 30 min of in situ warm ischemia followed by 24 h cold storage (UW solution) and subsequent 1 h ex vivo warm reperfusion.
4. DCD simvastatin group (DCD-Sim group): simvastatin pretreatment, DCD livers exposed to 30 min of in situ warm ischemia followed by 24 h cold storage (UW solution) and subsequent 1 h ex vivo warm reperfusion.

### 2.2. DCD Model and Liver Procurement

Rats were anesthetized by pentobarbital sodium (50 mg/kg) via intraperitoneal injection. After laparotomy, incision of the diaphragm resulted in cardiac arrest. Following 30 min warm ischemia in situ, the livers were perfused in situ with 60 ml 4°C cold heparinized (1 U/ml) saline via the aorta abdominalis to wash out the blood. The portal vein, superior hepatic caval vein and common bile duct were cannulated. Subsequently, the infrahepatic vena caval and the right adrenal veins were ligated. Then, livers were stored at 4°C in UW solution for 24 h.

### 2.3. The Isolated Perfused Rat Liver System (IPRL)

After 24 h cold storage, livers were exposed 15 min in the chamber at room temperature to simulate a rewarming period during liver implantation. Afterwards, livers were connected to the IPRL system for 60 min normothermic reperfusion ex vivo. Livers were reperfused through the portal vein only with freshly prepared Krebs-Henseleit bicarbonate buffer saturated with 95% O_2_ and 5% CO_2_. Flow velocity of perfusate was controlled at a flow rate of 15 ml/min [19] by a pulsatile perfusion pump (PING Technologies Inc., Shanghai, China). Portal venous pressure was measured continuously by a BL-420F pressure transducer system (Chengdu Taimeng Science and Technology Co., Chengdu, China) and adjusted to make sure that the portal venous pressure is below 8 mmHg. Samples of perfusate were collected via the superior hepatic caval vein catheter at different time points (5, 15, 30, and 60 min). After 1 h warm reperfusion, samples of perfusate, liver tissue, and bile output were collected for analyses.

### 2.4. Transaminases of Alanine Aminotransferase (ALT), Aspartate Aminotransferase (AST), and Bile Output Analysis

Hepatocyte injury was determined by detecting ALT and AST release in perfusate (the clinical laboratory of the Central South Hospital of Wuhan University) following the manufacturer's protocols. Liver function was measured by bile output (*μ*l of bile/g of liver) after ex vivo warm reperfusion.

### 2.5. Western Blotting Analysis

Liver samples were processed from −80°C storage and Western blotting performed as described [20]. The primary antibodies used are as follows: KLF2 (1 : 400, rabbit anti-KLF2 antibody, Biosynthesis Biotechnology, Beijing, China), phosphorylated eNOS at Ser1177 (1 : 1000, rabbit anti-phosphorylated eNOS antibody, Cell Signaling, Danvers, MA), total eNOS (1 : 1000, rabbit anti-eNOS antibody, Cell Signaling, Danvers, MA), Bax (1 : 1000, rabbit anti-Bax antibody, Proteintech, Manchester, UK), and Bcl-2 (1 : 1000, rabbit anti-Bcl-2 antibody, Proteintech, Manchester, UK). The bands were revealed by chemiluminescence ECL reagent (Proteintech, Manchester, UK), and protein expression was quantified by densitometric analysis using the Quantity One software package (Hertfordshire, UK). All bands were assayed for *β*-actin (1 : 1000, rabbit anti-*β*-actin antibody, Proteintech, Manchester, UK) content as standardization of sample loading.

### 2.6. Malondialdehyde (MDA), Superoxide Dismutase (SOD), and Adenosine Triphosphate (ATP) Analysis

For the evaluation of oxidative stress and mitochondrial function, frozen liver tissue was homogenized with butylhydroxytoluene-added Tris-HCl buffer determined by using the colorimetric assay kit specific for malondialdehyde (MDA), superoxide dismutase (SOD), and ATP (Nanjing Jiancheng Bioengineering Institute, Nanjing, China) following the manufacturer's instructions. The results were measured as *μ*mol/gprot.

### 2.7. Quantitative RT-PCR Analysis

Total RNA from frozen rat liver tissues was extracted using TRIzol reagent (Invitrogen Inc., Grand Island, NY, USA) and subsequent RNA was reverse-transcribed to cDNA (Thermo Scientific Revert Aid, USA) according to the manufacturer's instructions. SYBR green quantitative RT-PCR was used to assay the expression of target genes. The *β*-actin was detected as housekeeping gene. All primers were listed in Table 1.

### 2.8. Histopathology and TUNEL Staining

The paraffin-fixed liver sections were stained with hematoxylin-eosin (H&E). The liver tissue sections of IRI were graded blindly by Suzuki's criteria [21]. Histological changes were graded from 0 to 4 based on the degree of cellular vacuolization, hepatic sinusoid congestion, and hepatocyte necrosis.

Apoptosis was assayed with TUNEL staining (Roche Diagnostics, Indianapolis, IN, USA) according to the manufacturer's instructions. The total hepatocytes and TUNEL-positive cells were detected in 3 random chosen views (100x) for each liver sections using a fluorescence microscope. The rate of apoptosis (number of TUNEL-positive cells/total number of hepatocytes × 100%) in each view was measured with Image-Pro Plus 6.0 (Media Cybernetics, Rockville, MD, USA).

### 2.9. Statistical Methods

All data were analyzed using SPSS 16.0 statistical software for Windows (SPSS Inc., Chicago, IL, USA). All results are presented as the mean ± SD. One-way ANOVA was used to compare statistical significance between groups. Statistical significance was defined as *P* < 0.05.

## 3. Results

### 3.1. DCD Donor Pretreatment with Simvastatin Alleviates Hepatic IRI

To evaluate the effects of simvastatin pretreatment in DCD donors on hepatic injury derived from ex vivo warm reperfusion, hepatic function, bile production, hepatic architecture distortion, and presence of oxidative stress, inflammation, and apoptosis were measured.

**Figure 1:**
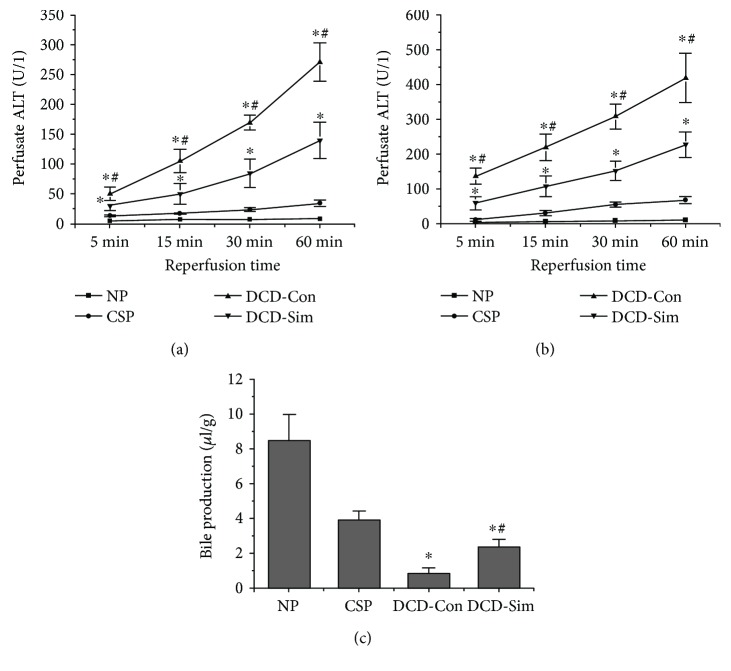
Simvastatin pretreatment alleviates hepatic IRI. Hepatic injury evaluated as release of transaminases (ALT and AST) in liver perfusate from rats at 5 min, 15 min, 30 min, and 60 min time points. Perfusate ALT (a) and ALT (b) levels were expressed as the mean ± SD (*n* = 6 per group; ^∗^*P* < 0.05 versus NP group, ^#^*P* < 0.05 versus DCD-Con group).

As shown in Figures 1(a) and 1(b), after donor cardiac arrest for 30 min in vivo and 24 h cold storage ex vivo, the levels of ALT and AST in the liver perfusate were remarkably increased at 5, 15, 30, and 60 min compared with those in the normal control group, while the peak was found at warm reperfusion for 60 min. However, DCD donor pretreatment with simvastatin significantly reduced the perfusate releases of ALT and AST at each time point compared with DCD control group (*P* < 0.05). As shown in Figure 1(c), after 30 min warm ischemia, 24 h cold-stored and 1 h warm-reperfused, the liver grafts released a lower quantity of bile in comparison to the normal control group. The detrimental effects were remarkably improved in DCD liver pretreatment with simvastatin (Figures 1(a), 1(b), and 1(c), *P* < 0.05).

### 3.2. DCD Donor Pretreatment with Simvastatin Ameliorates Hepatic Pathological Changes after Warm Reperfusion Subjected to 24 h Cold Preservation

**Figure 2:**
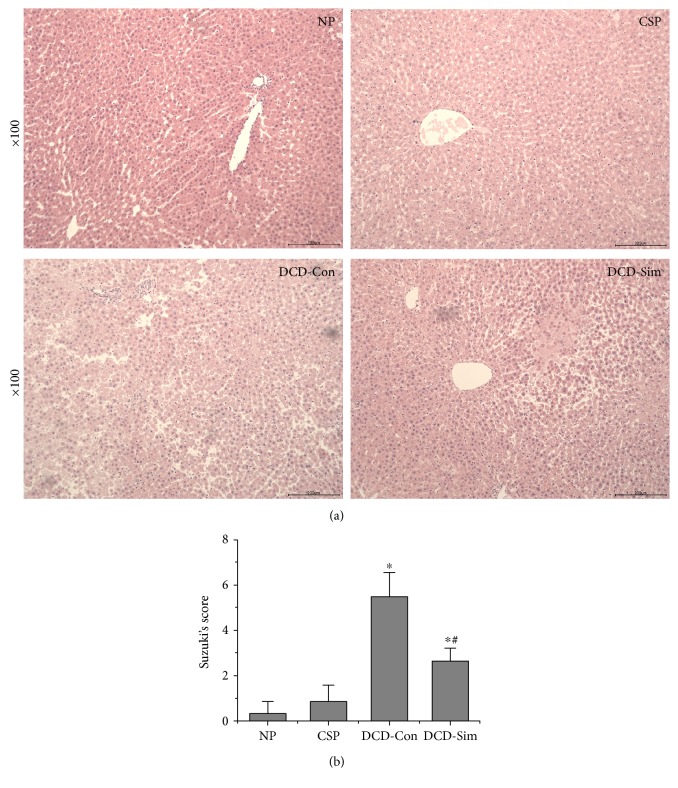
Simvastatin pretreatment ameliorates hepatic pathological changes. (a) Representative hematoxylin and eosin (HE) staining of liver tissues from rats after 1 h warm reperfusion. Original magnification, 100x. (b) Suzuki's histological score of liver tissue after 1 h warm reperfusion (*n* = 6; ^∗^*P* < 0.05 versus NP group, ^#^*P* < 0.05 versus DCD-Con group).

Figure 2(a) shows the same trends in H&E staining. Severe necrotic areas, cytoplasmic vacuolization of hepatocytes, and neutrophil infiltration were observed in the DCD control group, while DCD donor pretreatment with simvastatin groups showed minor injury after warm reperfusion. The results indicated that DCD donor pretreatment with simvastatin dramatically ameliorated pathological changes (*P* < 0.05, Figure 2(b)).

### 3.3. DCD Donor Pretreatment with Simvastatin Maintains the Expression of KLF2 and Its Protective Target Genes in Livers after Warm Reperfusion Subjected to 24 h Cold Preservation

**Figure 3:**
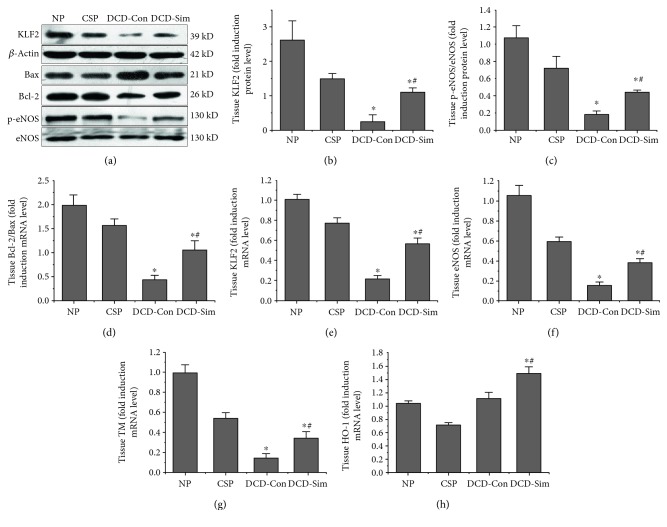
Simvastatin pretreatment maintains the expression of KLF2 and its protective target genes (a). The protein expression of KLF2 (b), phosphorylation eNOS (c), Bcl-2/Bax (d), and *β*-actin were determined by Western blotting and the gray values were calculated (*n* = 6 per group; ^∗^*P* < 0.05 versus NP group, ^#^*P* < 0.05 versus DCD-Con group). The mRNA expression of KLF2 (e), eNOS (f), TM (g), and HO-1 (h) were assessed by RT-PCR. The experiments were repeated three times and the data are shown as mean ± SD (*n* = 6; ^∗^*P* < 0.05 versus NP group, ^#^*P* < 0.05 versus DCD-Con group).

After donor cardiac arrest for 30 min in vivo and 24 h cold storage ex vivo, the rat livers exhibited a significant reduction in KLF2 protein and mRNA expression after warm reperfusion compared with the normal control livers (Figures 3(b) and 3(e)). Meanwhile, DCD control group showed significant decreases in the expression of KLF2's protective target genes, such as eNOS (phosphorylation protein and mRNA levels, Figures 3(c) and 3(f)), TM (Figure 3(g)), and HO-1 (Figure 3(h)). DCD donor pretreatment with simvastatin effectively prevented the decay of liver KLF2, eNOS, TM, and HO-1 after warm reperfusion subjected to 24 h cold preservation (Figures 3(a), 3(b), 3(c), 3(d), 3(e), 3(f), and 3(g), *P* < 0.05).

### 3.4. DCD Donor Pretreatment with Simvastatin Reduces Oxidative Stress and Improves ATP Levels in Livers after Warm Reperfusion Subjected to 24 h Cold Preservation

**Figure 4:**
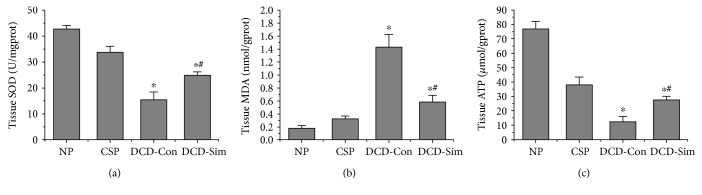
Simvastatin pretreatment reduces oxidative stress and improves ATP levels. (a) Liver tissue release of SOD level, (b) liver tissue release of MDA level, and (c) liver tissue release of ATP level (*n* = 6; ^∗^*P* < 0.05 versus NP group, ^#^*P* < 0.05 versus DCD-Con group).

As shown in Figures 4(a) and 4(b), after donor cardiac arrest for 30 min in vivo and 24 h cold storage ex vivo, the rat livers in DCD control group showed a severe increase in oxidative stress after warm reperfusion compared with the normal control livers. Moreover, DCD liver pretreatment with simvastatin significantly increased SOD and reduced MDA levels in comparison with DCD control group (*P* < 0.05). Furthermore, as shown in Figure 4(c), DCD liver pretreatment with simvastatin significantly increased ATP levels after warm reperfusion in comparison with DCD control group, but were still significantly lower than the one in the CSP group (*P* < 0.05).

### 3.5. DCD Donor Pretreatment with Simvastatin Inhibits Innate Immune Responses and Inflammation in Livers after Warm Reperfusion Subjected to 24 h Cold Preservation

**Figure 5:**
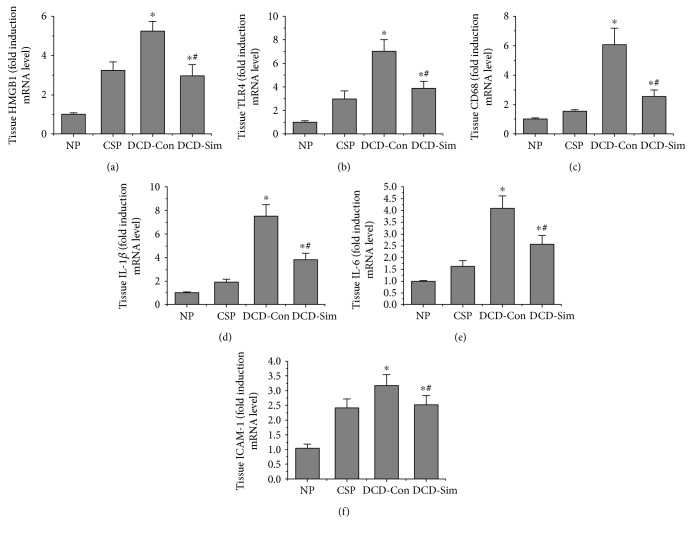
Simvastatin pretreatment inhibits innate immune responses and inflammatory. The mRNA expression of HMGB1 (a), TLR4 (b), CD68 (c), IL-1*β* (d), IL-6 (e), and ICAM-1(f) were assessed by RT-PCR. The experiments were repeated three times and the data are shown as mean ± SD (*n* = 6; ^∗^*P* < 0.05 versus NP group, ^#^*P* < 0.05 versus DCD-Con group).

As shown in Figures 5(a), 5(b), and 5(c), DCD liver pretreatment with simvastatin significantly inhibited high-mobility group box 1 (HMGB1), Toll-like receptor 4 (TLR4), and CD68 mRNA levels after warm reperfusion in comparison with DCD control group, but were still significantly more than the normal control group (*P* < 0.05). In addition, DCD donor pretreatment with simvastatin pretreatment significantly reduced the levels of inflammatory cytokines. Real-time PCR also was used to evaluate the mRNA expression of these inflammatory factors. As shown in Figures 5(e), 5(f), and 5(g), compared with the DCD control group, simvastatin pretreatment dramatically suppressed the mRNA expression of interleukin-1*β* (IL-1*β*), IL-6, and intercellular adhesion molecule 1 (ICAM-1). These results provided strong evidence that DCD liver pretreatment with simvastatin could significantly reduce the release of HMGB1, TLR4, CD68, IL-1*β*, IL-6, and ICAM-1 after warm reperfusion subjected to 24 h cold preservation in rats.

### 3.6. DCD Donor Pretreatment with Simvastatin Suppresses Hepatocyte Apoptosis in Livers after Warm Reperfusion Subjected to 24 h Cold Preservation

**Figure 6:**
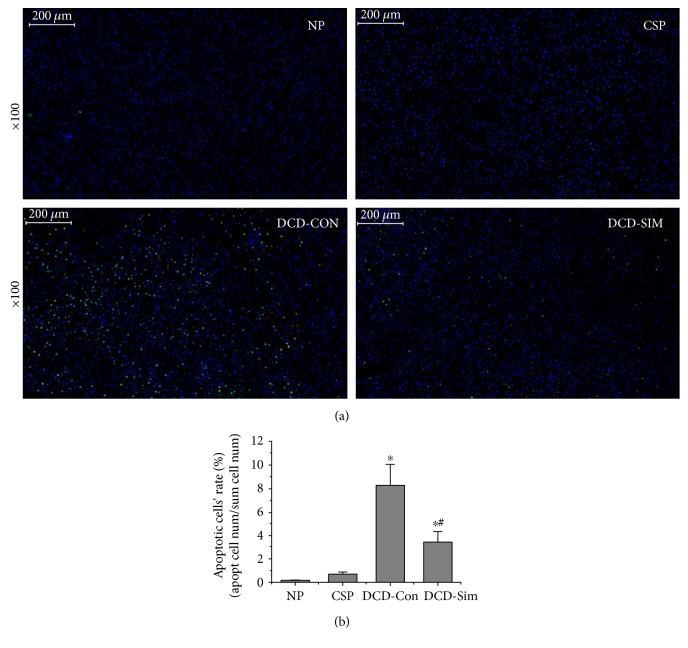
Simvastatin pretreatment suppresses hepatocyte apoptosis. (a) Representative images of TUNEL staining determined in livers from rats after 1 h warm reperfusion. Original magnification, 100x. (b) Quantitative analysis of liver apoptosis cells (*n* = 6; ^∗^*P* < 0.05 versus NP group, ^#^*P* < 0.05 versus DCD-Con group).

Bax and Bcl-2 are important markers of apoptosis. Bax is a proapoptotic protein, while Bcl-2 is an antiapoptotic protein. Western blotting was used to investigate the expression of markers of apoptosis at the protein level (Figures 3(a) and 3(c)). The results showed that Bcl-2/Bax (ratio) was downregulated in livers of the DCD control group and upregulated in DCD liver pretreatment with simvastatin group after warm reperfusion subjected to 24 h cold preservation (*P* < 0.05). As shown in Figure 6(a), the results of TUNEL staining demonstrated that a large amount of apoptotic cells were detected in the DCD control group and few apoptotic cells were seen in the DCD liver pretreatment with simvastatin group. It indicated statistically significant differences between the two groups by Image-Pro Plus software (Figure 6(b), *P* < 0.05).

## 4. Discussion

Hepatic IRI injury is a complex pathological process associated with liver transplantation, shock, and trauma. The IRI is a serious threat for short- and long-term graft outcome of liver transplantation. Moreover, DCD livers have a unique process of hepatic IRI, which includes severe warm ischemia before organ procurement in vivo, more sensitive cold ischemia during the period of organ preservation in vitro, and reperfusion injury after transplantation in vivo. Unfortunately, the therapeutic strategies to improve IRI in DCD livers are currently limited. Importantly, recent researches have demonstrated that donor simvastatin pretreatment can protect against hepatic IRI in livers from healthy, cirrhotic, and obese animals [15, 17, 22–25]. Therefore, the purpose of this study was to determine whether DCD donor simvastatin treatment ameliorates warm and cold ischemia-induced injury in liver allografts subjected to 24 h cold preservation. The major novel findings that emerged from our experiments are as follows: (1) Donor simvastatin pretreatment improves DCD liver quality after reperfusion in rats. (2) The protective effect of simvastatin is mediated via induction of KLF2, eNOS, TM, and HO-1 to improve hepatic microcirculation. (3) DCD liver simvastatin pretreatment can inhibit innate immune responses particularly via suppressing TLR4 and alleviating the release of HMGB1. (4) DCD liver simvastatin pretreatment reduces release of mitochondrial ROS to prevent oxidative stress. (5) DCD liver simvastatin pretreatment inhibits release of inflammatory cytokines.

In the present study, we had well established the model of DCD livers in rats. Our results showed that DCD donor pretreatment with simvastatin significantly decreased perfusate levels of AST and ALT compared with those in the DCD control group at 1 h reperfusion subjected to 24 h cold preservation (Figures 1(a) and 1(b)). These results were consistent with the pathological changes (Figures 2(a) and 2(b)). Extensive hepatocyte necrosis, cytoplasmic vacuolization of hepatocytes, and neutrophil infiltration of liver tissues were observed in the DCD control group, which was obviously ameliorated in the simvastatin treatment group. In addition, bile production was markedly increased in the simvastatin treatment group compared to the DCD control group (Figure 1(c)). Our results demonstrated that simvastatin pretreatment attenuated perfusate liver enzyme levels and pathological changes and improved synthetic function in DCD livers after reperfusion.

The process of IRI in DCD livers are generally viewed as a serious event comprising the loss of oxygen supply and metabolic substrates during warm ischemia in vivo and preservation in vitro and the production of ROS and production of inflammatory mediators during reperfusion of blood flow. Moreover, during the process of organ hypoxia and reoxygenation, loss of blood pulsatile flow and vascular mechanical shear stress during allograft warm ischemia and preservation has been shown to play critical roles in IRI. Our results first demonstrated that DCD donor pretreatment with simvastatin had a better effect on functional recovery of DCD livers. However, the underlying mechanism of simvastatin in DCD liver IRI remains unclear. Indeed, recent studies have reported that flow cessation of the biomechanical stimulus by shear stress deteriorated the endothelial barrier homeostasis [15, 26] and activated proinflammatory [27] and prothrombotic pathways [27] by downregulating the expression of transcription factor KLF2. The present study showed that DCD livers after ex vivo reperfusion injury subjected to 24 h cold preservation downregulated the expression of KLF2 and p-eNOS (protein and mRNA levels), while DCD donor pretreatment with simvastatin upregulated the expression of KLF2 (protein and mRNA levels), eNOS (protein and mRNA levels), HO-1 (mRNA levels), and TM (mRNA levels) (Figures 3(a), 3(b), 3(c), 3(e), 3(f), and 3(g)). These observations demonstrated that the protective effects of simvastatin treatment in DCD donors may be due to the induction of the shear stress-regulated KLF2 and eNOS [9, 28].

Furthermore, several pathways have been demonstrated to be associated with KLF2, including eNOS pathway, HO-1/ROS/Bcl-2 pathway, HMGB1/TLR4/NF-kappab pathway and inflammatory pathway. Hypoxia triggers the release of ROS and HMGB1 in different compartment molecules during procurement, preservation, and implantation [29, 30]. As the production of HMGB1 depends on the quantity of intracellular ROS, mitochondria was regarded as the source of the oxidative stress [30–33]. Therefore, it has recently been suggested that reduction of the initial release of ROS and HMGB1 molecule in donors could be important for subsequent prevention of immune and inflammatory responses [32, 34, 35]. In our study, we found that DCD donor pretreatment with simvastatin downregulated the mRNA expression of HMGB1 and TLR4 in the liver after 1 h reperfusion subjected to 24 h cold preservation compared to the DCD control group (Figures 5(a), 5(b), and 5(c)). In accordance with mitochondria function, the release of SOD increased and MDA levels of liver tissue lowered in DCD donor pretreatment with simvastatin group after reperfusion ex vivo compared to DCD control group (Figures 4(a) and 4(b)). This also might be attributed to the antioxidant properties of KLF2 through activation of nuclear factor erythroid-derived 2-like 2 (Nrf2) and upregulation expression of HO-1 [36, 37]. Next, the liver ATP was better functionally recovered in simvastatin group (Figure 4(c)). To explore the antiapoptotic or apoptotic mechanism of simvastatin on DCD livers, we evaluated the expression of Bcl-2 and Bax protein in liver tissue. Our results showed that DCD livers after ex vivo reperfusion injury subjected to 24 h cold preservation upregulated Bax protein and downregulated Bcl-2 protein while pretreatment with simvastatin significantly increased the expressions of Bcl-2 protein and downregulated the expression of Bax protein (Figures 3(a) and 3(c)). With TUNEL assay, DCD livers pretreated with simvastatin showed a significant lower apoptotic cell ratio, as compared with DCD control group (Figures 6(a) and 6(b)).

Finally, we investigated the expression of IL-1*β*, IL-6, and ICAM-1 at the mRNA levels. Our results showed that DCD donor pretreatment with simvastatin significantly decreased the levels of these inflammatory cytokines compared with those in the DCD control group (Figures 5(e), 5(f), and 5(g)), and thus the changes might also be depended on the anti-inflammatory potential of KLF2 [27, 38]. In turn, proinflammatory cytokines such as IL-1*β* also have been shown to repress KLF2 expression [39]. These observations demonstrated that the anti-inflammatory protective effects of DCD donor pretreatment with simvastatin reduced the neutrophil adhesion and extravasation.

Importantly, regarding the underlying mechanism on how KLF2 activators improve liver quality, Guixe et al. firstly suggested that simvastatin activated the upregulation of Rab7 and autophagic flux in the cold-stored liver sinusoidal endothelium [18]. Our results were consistent with the above findings and indicated the protective effects of simvastatin. It requires further research to explore the cross-talk between KLF2 and autophagy on DCD livers. Nevertheless, there were also some limitations in our study. Firstly, we used the ex vivo isolated liver reperfusion model to evaluate DCD liver quality but not the liver transplantation, which is closer to physiology. Next, the role of KLF2 in protection against DCD liver IRI could be further illustrated using genetic tools where KLF2 expression would be overexpressed or knocked down specifically.

In conclusion, the present study first demonstrates that DCD donor pretreatment with simvastatin improved DCD livers' functional recovery shortly after ex vivo reperfusion subjected to 24 h cold preservation and strongly protects liver cells. The protective mechanisms of simvastatin-reduced DCD liver IRI might be mediated via upregulation of the flow-dependent protective genes KLF2 conferring anticoagulant, suppressing innate immune responses, antioxidant, antiapoptotic, and anti-inflammatory properties. Our results strongly supported pretreating potential DCD donors with simvastatin as a feasible strategy for liver organ protection. Ultimately, this possibility will require further clinical trials to evaluate the protective effects of statins in liver transplantation.

## Figures and Tables

**Table 1:** Nucleotide sequences of primers used for quantitative RT-PCR.

Gene		Primer sequence (5′—3′)
KLF2	Forward	GAGCCTATCTTGCCGTCCTT
	Reverse	AGCACGCTGTTTAGGTCCTC

eNOS	Forward	CAACTGGAAAAAGGCAGCCC
	Reverse	AAGAGCCTCTAGCTCCTGCT

TM	Forward	CCTTTGTCTTTCCGGGCTCT
	Reverse	TCAAGTCCTCCCTACCCTCG

HO-1	Forward	CCTGCTAGCCTGGTTCAAGA
	Reverse	GAGTGTGAG GACCCATCGCA

HMGB1	Forward	GGCGGCTGTTTTGTTGACAT
	Reverse	ACCCAAAATGGGCAAAAGCA

TLR4	Forward	TGTATCGGTGGTCAGTGTGC
	Reverse	CAGCTCGTTTCTCACCCAGT

CD68	Forward	CGTTACCCGGAGACGACAAT
	Reverse	TCCTTGGTGGCCTACAGAGT

IL-1*β*	Forward	GACTTCACCATGGAACCCGT
	Reverse	GGAGACTGCCCATTCTCGAC

IL-6	Forward	AGAGACTTCCAGCCAGTTGC
	Reverse	AGTCTCCTCTCCGGACTTGT

ICAM-1	Forward	GCAGGTGAACTGCTCTTCCT
	Reverse	GTCTTCCCCAATGTCGCTCA

*β*-Actin	Forward	TGCTATGTTGCCCTAGACTTCG
	Reverse	GTTGGCATAGGTCTTTACGG
